# In vitro assessment and comparison of quality of alcohol-based hand rubs, pre- and peri-COVID-19 pandemic outbreak in Kenya

**DOI:** 10.12688/f1000research.140226.2

**Published:** 2024-04-30

**Authors:** Samuel Omari, Florence Ng'ong'a, James Kimotho

**Affiliations:** 1 Department of Biochemistry, Jomo Kenyatta University of Agriculture and Technology, Nairobi, Kenya; 2 Innovation and Technology Transfer Division, Kenya Medical Research Institute, Nairobi, Kenya

**Keywords:** Coronavirus, COVID-19, Pandemic, Alcohol-based hand sanitizers, hand hygiene, Infection prevention

## Abstract

**Background:**

In the wake of the coronavirus disease 2019 (COVID-19) pandemic, the World Health Organization recommended the use of alcohol-based hand rubs (ABHRs) to curb transmission, leading to increased production and use. This has posed a danger of production and use of poor-quality ABHRs.

**Methods:**

This study assessed and compared the quality of ABHRs in the Kenyan market that were produced before and after the outbreak of the COVID-19 pandemic in March 2020. Quality testing was carried out against European EN 1500:2013 and Kenyan EAS 789:2013 Standards.

**Results:**

The study found that 27.8% of the peri-pandemic sanitizers had less than 90% bactericidal reduction activity as compared to 12.5% manufactured pre-pandemic. Only 25% peri-pandemic ABHRs met the EAS 789:2013 acceptable limit of over 60% alcohol content. Product adulteration with methanol was found in 20 % of the samples with only 5% complying with FDA approval limit of <0.063% v/v methanol. Study found no correlation between the total alcohol content and the efficacy of ABHRs.

**Conclusions:**

The study found that more substandard products were produced during the pandemic. This raises a concern about possible emergence of alcohol resistant strains of microorganisms. The study therefore recommends an adequate quality monitoring system to curb against substandard products.

## Introduction

In the wake of the coronavirus disease 2019 (COVID-19) pandemic, the World Health Organization (WHO) widely recommended hand hygiene as one key containment strategy to the spread of the virus, which included frequent hand washing with soap and water and the use of alcohol-based hand rubs (ABHRs) with more than 60% alcohol content. ^1^ A 30-second application of ABHRs has been reported to have a better disinfection efficacy than traditional soap and water approaches, with more than 3.5 log10 reduction in bacterial counts. ^2^ Two ABHRs formulations had subsequently been recommended by WHO, one containing 80% ethyl alcohol and another formulation containing 75% isopropyl alcohol. ^3^ The use of sanitizer therefore has been embraced in both the hospital environment and community to prevent acquired infections (HAIs and CAI, respectively). ^4^

In March 2020, the Kenyan government, as part of the measures adapted to curb spread of COVID-19, also recommended the use of ABHRs. This resulted in an increased production of different brands in the Kenyan market ^5^ and their widespread use, which in turn increased the possible development of resistant strains of microorganisms. ^6^ Exposure of microorganisms to inappropriately used or poor-quality ABHRs can lead to the survival of some strains, subsequently leading to resistance. ^6^^–^^9^ Such resistance has been reported in *Enterococcus faecium* as well as *Salmonella typhimurium.* ^10^^,^^11^ Carrying out studies on the quality and effectiveness of these brands of ABHRs as well as the emerging resistance due to poor-quality ABHRs is imperative.

The aim of this study was to assess and compare the quality and efficacy of alcohol-based hand rubs against *Escherichia coli* and *Staphylococcus aureus* pre- and peri-COVID-19 pandemic outbreak in Kenya.

## Methods

### Sample collection

**Figure 1.**
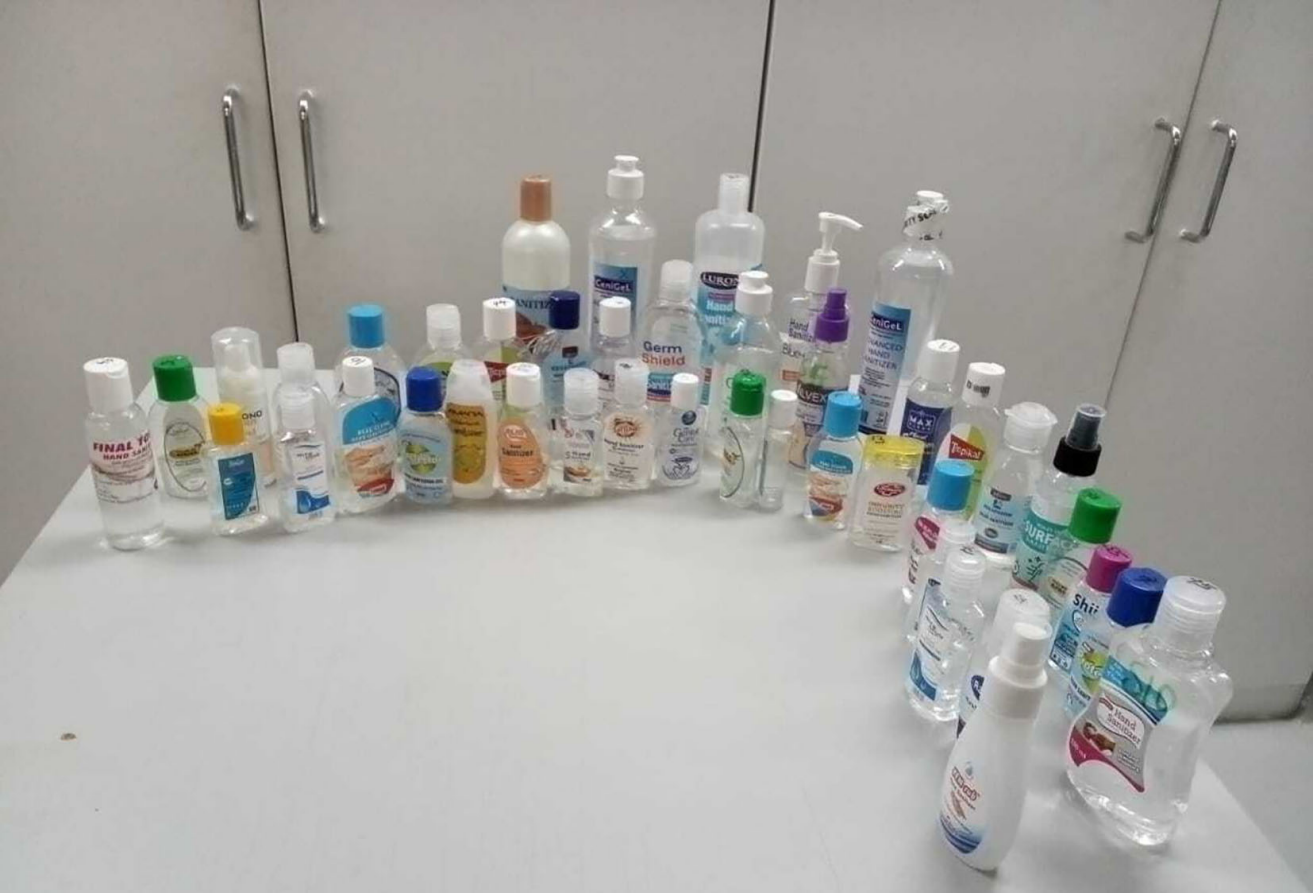
Samples of brands of alcohol-based hand rubs used in this study.

A total of 90 ABHRs samples were collected for the study period through convenience sampling method, as the total number of ABHRs brands in the Kenyan market was not known. The KEMRI Innovation & Technology Division (KITTD) had collected ABHRs from shelves and archived them as part of its research and development activities before March 2020. A total of 55 archived sanitizers within the expiration period at the time of laboratory testing were selected for analysis. In addition, 35 different sanitizer brands from the local retail shelves manufactured during the pandemic period, *i.e.* after March 2020 to June 2021, were collected as peri-COVID-19 samples ( Figure 1) *.* During the peri-pandemic sanitizer sample collection highest priority was given to the brands found to have been already collected and archived during the pre-pandemic period. They were cross-checked for regulatory compliance using the Kenya Bureau of Standards USSD verification database. ^12^ All hand sanitizers collected from the market were tested. The products were visually inspected for labelling compliance on declaration of active ingredient and against the parameters specified in the KS EAS 789:2013 standard. In addition, control samples were prepared in the KITTD Laboratory using protocol as described by WHO for quality assurance so as to ensure validity and reliability of results obtained during efficacy testing. ^3^

### Alcohol content assay and pH of alcohol-based hand rubs

The assay profiled and quantified three analytes: ethanol, isopropyl alcohol and methanol using Shimadzu GC-2010 plus - Gas Chromatography with Flame Ionization Detector (GC-FID) following the protocol as described by Zhang. ^13^ This was done as follows: the working solution (WS) was prepared by diluting glycerin in distilled water to a concentration of 4.6% (v/v). The alcohol calibration standards were prepared by adding aliquots of pure alcohols - ethanol, IPA, methanol, n-propanol (n-PA) - to distilled water. The internal standard (IS) acetonitrile (ACN) was added to the calibration solutions at a concentration of 5% (v/v). The quality control sample was prepared by diluting 25 μL of ethanol, 25 μL of IPA, and 50 μL of acetonitrile to 1 mL in distilled water. The gel ABHR samples were diluted before injection due to viscosity. These samples were analyzed in the GC-FID with the following analytical parameters:

• Split/Splitless inlet: 250°C, split ratio 20:1
• Injection volume: 0.2 μL
• Carrier gas: helium
• Column flow rate: 7 mL/min, constant flow mode
• Oven: 50°C (5 min), 30°C/min to 230°C (3 min)
• FID: 250°C, air: 400 mL/min, fuel gas (H _2_): 30 mL/min, constant make up flow: 18 mL/min

Determination of pH of ABHRs samples was measured using Thermo Scientific Orion Star A214 pH and ISE Benchtop Meter with limits as specified by KS EAS 789:2013 standard.

### Alcohol-based hand rubs efficacy testing

Efficacy testing was carried out using quality control strains of *E. coli* ATCC 25922 and *S. aureus* ATCC 25923 as described in the European Standard (EN) 1500:2013 ^14^ and KS EAS 789:2013. Briefly, 0.5 Mac Farland suspensions of the microorganisms were separately prepared as per the method described by the Clinical and Laboratory Standards Institute. ^15^ As a quality control measure, so as to ensure validity and reliability of results obtained during efficacy testing, a control sample of 80% ethyl alcohol was prepared from absolute ethanol by mixing 800 mL of pure ethyl alcohol and 200 mL of distilled as guided by WHO ^3^; this was further tested using an alcoholmeter to measure the alcohol percentage in the control sample. This was tested together with the ABHR samples. Means of the colony forming units were used in determining log reduction values.

### Determination of logarithmic reduction

Logarithmic reduction factors (RF) were assessed based on pretreatment and post-treatment with the ABHRs and the results of each ABHRs manufactured during the pandemic period compared to those collected before the pandemic. The logarithmic reduction factors were expressed as a percent reduction. Log reduction was calculated as log10 (pretreatment A) - log10 (post treatment B) and the percent reduction was calculated as (A-B)/A% where; where A = number of viable microorganism at before treatment and B = number of viable microorganism after treatment. ^14^

## Results

### Declaration of active ingredients

**Figure 2.**
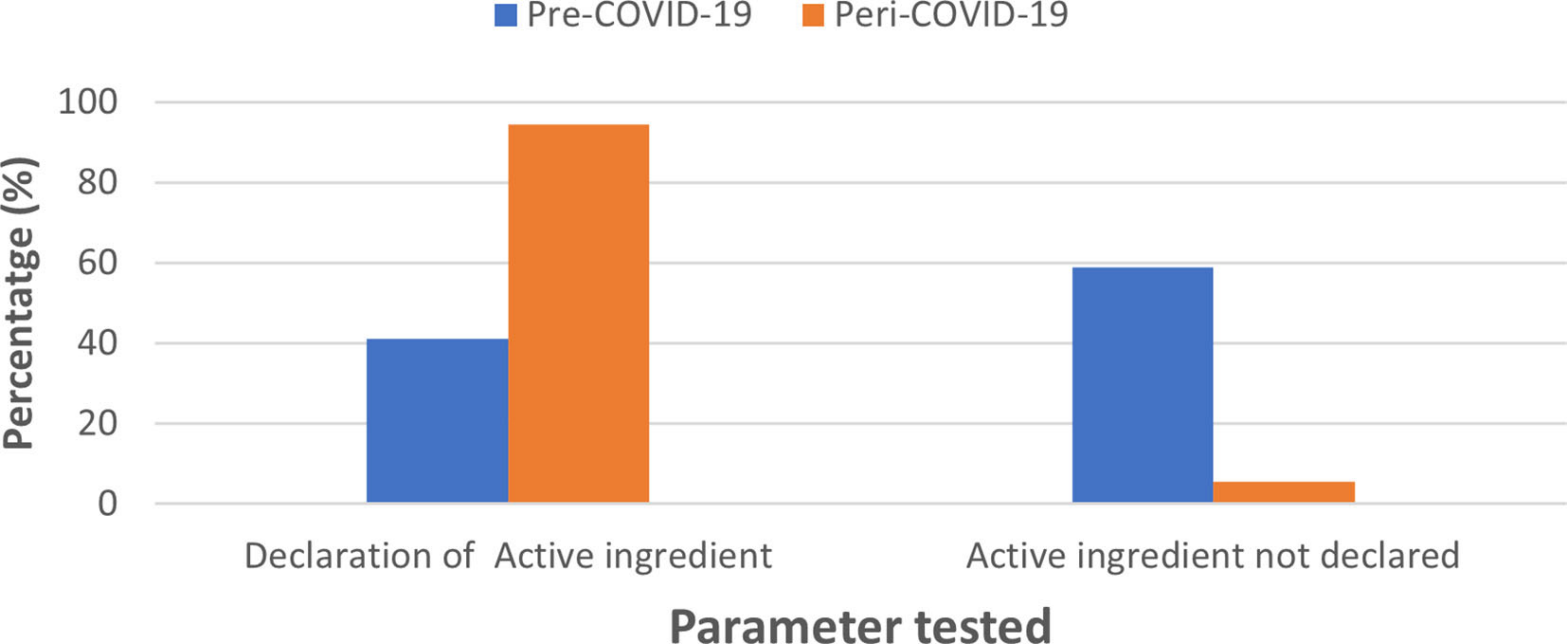
Comparison of standards in labelling ingredients for pre-COVID-19 and peri-COVID-19 periods.

In the case of active ingredients, 41.08% of pre-COVID-19 sanitizers listed the active ingredient used; of these, 16.08% specifying the percentage composition of active ingredient which included ethanol, isopropyl alcohol (IPA) and 25% did not indicate the exact percentage composition. A total of 58.9 % did not list the active ingredient. For sanitizer samples collected in the peri-pandemic period, the majority of the brands (94.4%) listed the active ingredients used; of these, 66.7% listed >60%, alcohol as an active ingredient and 27.7% did not declare their exact percent alcohol content; sanitizers that did not declare the active ingredient composition accounted for 5.56% ( Figure 2).

### Shelf lives of the ABHRs

**Figure 3.**
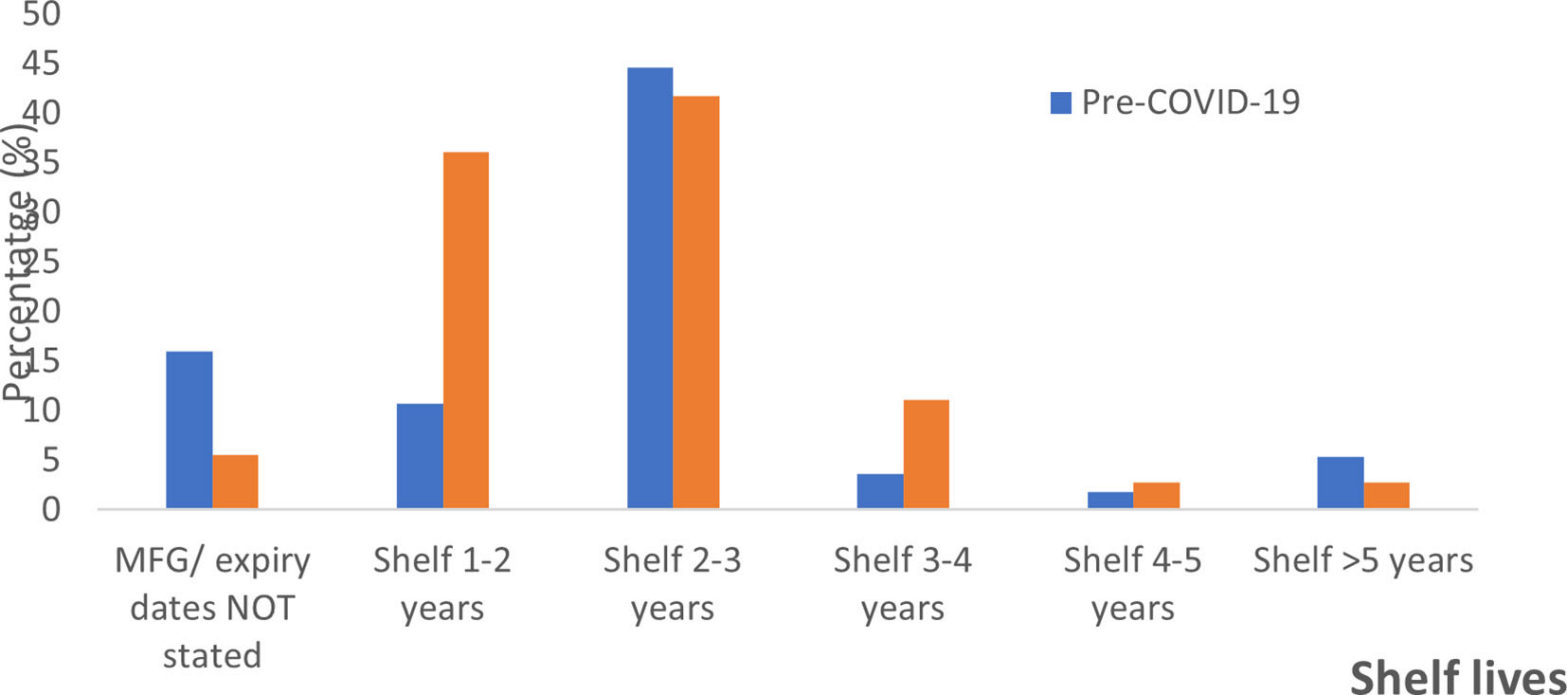
Percentage distribution of shelf life for sanitizers before and during the COVID-19 pandemic.

The shelf lives allocated to the sanitizers were highly varied and ranged from one to five years. For pre-COVID-19 alcohol-based hand rubs, 16% did not state the manufacture and/or expiry dates, as compared to only 5.56% (n = 2) for the peri-pandemic period. The majority of the hand sanitizers manufactured during either of the periods under review had shelf lives of between two-three years, *i.e.* 44.6 % for pre-COVID and 41.7% for peri-pandemic ABHRs. Shelf life was one-two years (10.7%), three-four years (3.6%), four-five years (1.79%), more than five years (5.36%) for pre-pandemic ABHRS and one-two years (36.1%), three-four years (11.1%), four-five years (2.8%), and more than five years (2.8%) for peri-pandemic ABHRs ( Figure 3). The manufacture period for the pre-COVID samples ranged from September 2017 to March 2020 and the study sorting period for pre-COVID-19 sanitizer was capped to March 2020. The manufacturer period for peri-COVID-19 ABHRs samples ranged from March 2020 to April 2021.

### Efficacy performance of ABHRs

Of the total pre-COVID-19 sanitizers tested, 12.5% (n = 7) had a performance of less than 1 log reduction, as compared to 27.8% (n = 10) peri-COVID-19 ABHRs which had a performance of less than 1 log reduction. Overall, 78.6% had between 1-6 log reduction with two samples including quality control sample displaying total microbial reduction, while most of the ABHRs manufactured during the pandemic (69.4%) showed between 1-3 log reduction with no sample manufactured during the pandemic period showing an efficacy performance above 3 log reduction ( Table 1). These results display a reduction in quality performance of the ABHRs manufactured during the pandemic period in terms of efficacy.

**Table 1.** Comparison of ABHRs efficacy performance pre- and during the COVID-19 pandemic.

Log reduction	Frequency pre-COVID-19	Percentage	Frequency peri-COVID-19	Percentage
<1	7	12.5	10	27.8
1 to 2	31	55.4	21	58.3
2 to 3	11	19.6	4	11.1
3 to 4	2	3.6	0	0
5 to 6	3	5.4	0	0
>6	2	3.6	1	2.8
Total	56	100	36	100

**Figure 4.**
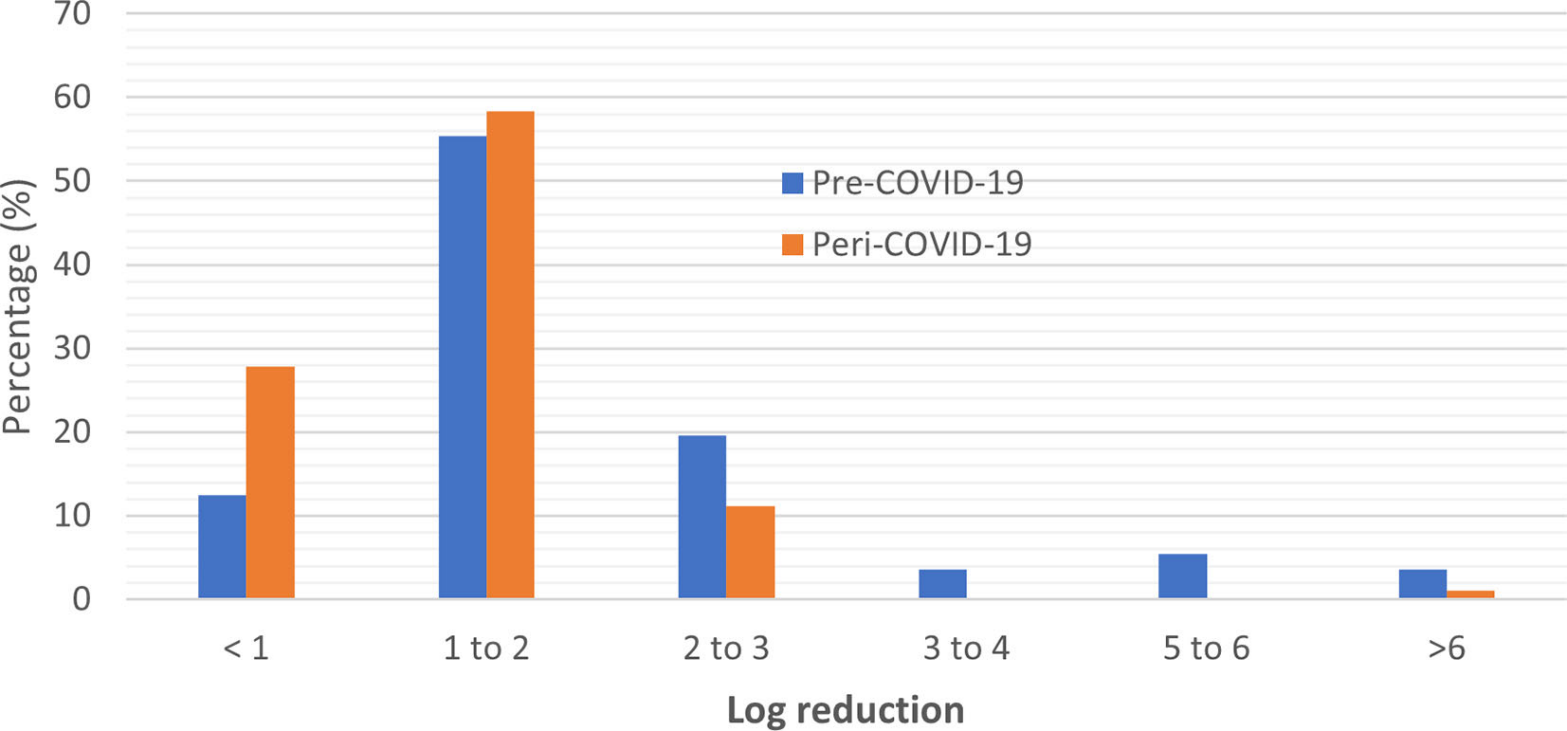
Distribution of sample comparison according to their log reduction.

Based on the distribution analysis for the general performance of the ABHRs, pre and peri pandemic, there was an observed clustering of the performance between <1 to 3 with an unexpected gap between 3-5 logarithmic units for peri-pandemic ABHRs ( Figure 4). This is a strong positive correlation of log reduction between pre-COVID and peri-COVID ABHRs samples (Pearson correlation co-efficient value of R was 0.9328. The p-value was < 0.00001 implying that the result was significant at p < 0.05. This is attributed to the increased pre-COVID log reduction of the values above 3.

### Alcohol content assay and pH of ABHRs

A total of 20 peri-COVID samples were analyzed using gas chromatography with flame ionization detector (FID). The assay profiled and quantified three analytes: ethanol, isopropyl alcohol and methanol. pH was also measured as specified by KS EAS 789:2013 standard. Five samples (25%) complied and had > 60% alcohol content ( Table 2). Sample PBHR 2, PBHR 9, PBHR 16 and PBHR 25 had been labelled by the manufacturer as containing IPA. However, upon analysis the first three contained ethanol as the active component while the fourth product contained a mixture of ethanol (30.41%) and methanol (31.12%).

**Table 2.** Analytical results for alcohol-based hand rubs.

Sample code	Alcohol concentration (percentage v/v)			pH	Failed tests
	Ethanol content %v/v	IPA content %v/v	Methanol content %v/v		
PBHR 02	72.46	ND	2.72	6.7	M
PBHR 04	0.07	0.18	25.82	8.6	A, *M, P
PBHR 06	34.75	ND	42.21	5.4	A, *M, P
PBHR 08	79.09	ND	0.1	5.8	M, P
PBHR 09	68.95	ND	0.06	8.6	P
PBHR 11	47.21	ND	0.07	6	A, M
PBHR 13	47.45	10.93	0.08	6.1	A, M
PBHR 15	67.92	ND	0.07	6.6	M
PBHR 16	63.45	ND	4.64	5.7	M, P
PBHR 17	40.8	ND	0.08	6.8	A, M
PBHR 20	54.49	ND	0.11	5.7	A, M, P
PBHR 21	10.79	11.07	34.26	7.6	A, *M
PBHR 22	40.35	ND	0.08	5.5	A, M, P
PBHR 23	52.23	ND	2.05	6.9	A, M
PBHR 25	30.41	2.74	31.12	5.5	A, *M, P
PBHR 26	56.56	ND	0.07	6.4	A, M
PBHR 30	34.78	ND	0.07	5.5	A, M, P
PBHR 33	38.25	ND	0.07	5.6	A, M, P
PBHR 34	50.7	ND	2	7	A, M
PBHR 36	57.94	ND	0.12	0.4	A, M, P

A – Alcohol assay, IPA – Isopropyl alcohol, *M – Methanol substitution, M – Methanol limit, ND – Not detected, P – PH (limits 6-8 in the KEBS standard).

**Figure 5.**
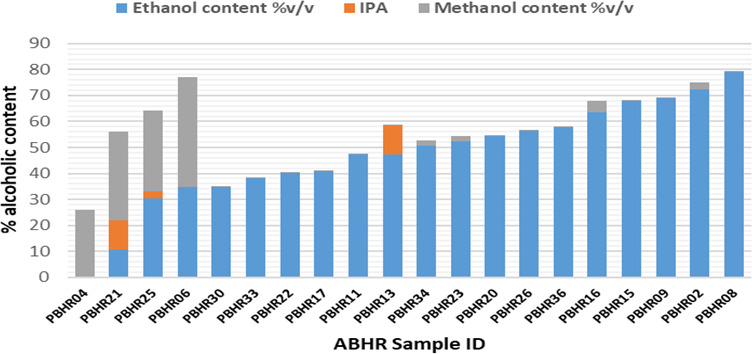
Alcohol content in ABHRs.

Only five samples (25%) had an alcohol content of above 60% v/v which is the KEBS minimum limit for alcohol content in hand sanitizers. No sample met the required content of the World Health Organization (WHO) of 80% v/v of ethanol ( Figure 5).

**Figure 6.**
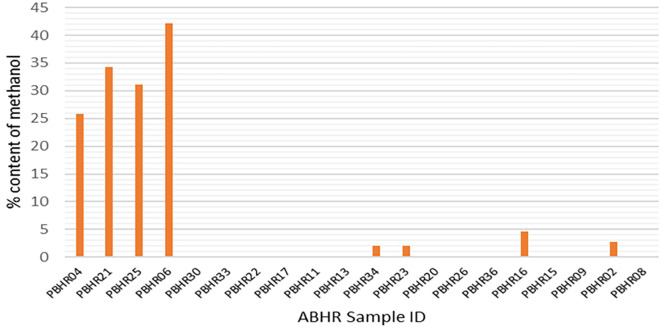
Methanol content in ABHRs.

The FDA-accepted limit of methanol is < 0.063, ^16^ however, only one ABHR (ABHR 09) met the FDA requirement with a methanol content of 0.06%; meanwhile, methanol substitution was found in four samples (20%), indicative of impure ethanol being used in the production process ( Figure 6). The variation of the content of methanol was high (7.29 ± 6.003) at a 95% confidence limit.

**Figure 7.**
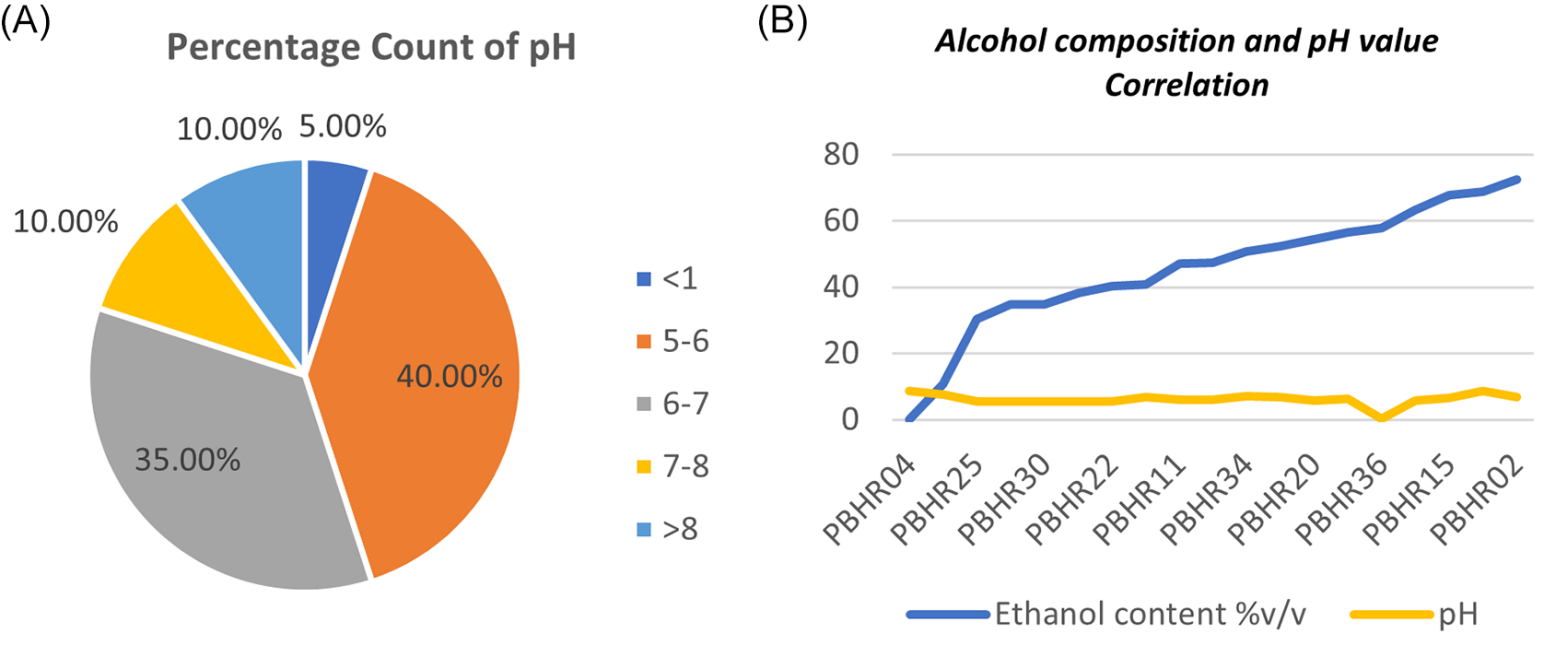
(A) Percentage count of pH distribution among the products; (B) Correlation between alcohol composition and pH value.

One ABHR sample had a major deficiency that resulted in outlier pH results: P-ABHR 36 showed an acidic pH of 0.4 pH units. Most (55%, n = 12) samples had pH above the minimum limit of 6, with the range being 0.4–8.6 pH units ( Figure 7A). The study found no correlation between the pH of a product and the total content of alcohol in tested the samples (Coefficient correlation R was -0.1078 and p-value was 0.65) ( Figure 7B).

**Figure 8.**
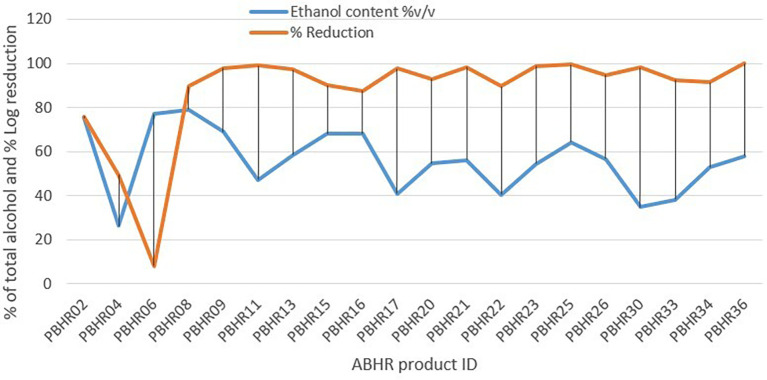
Correlation between ethanol content and percentage reduction.

Unexpectedly, results of the Pearson correlation analysis indicated that there was a non-significant relationship between total alcohol content and percentage log reduction, (r = - 0.167, p = 0.480) ( Figure 8).

### Impact of the Kenya Bureau of Standard mark of product quality

**Figure 9.**
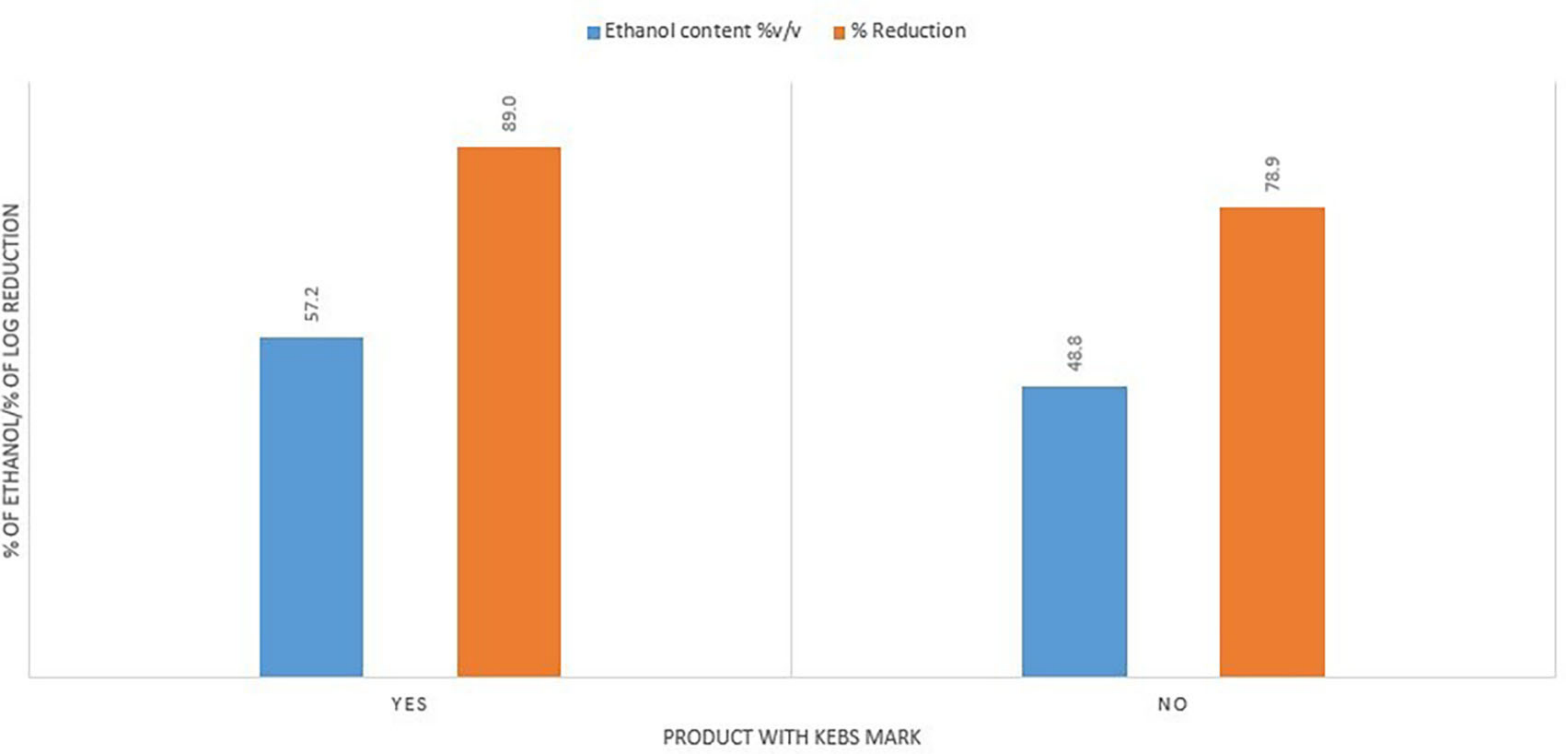
Ethanol content and % reduction comparison between KEBS certified and uncertified ABHRs.

The Kenya Bureau of Standards had a positive impact on the quality of the ABHRs. ABHRs with a KEBS standardization mark had a higher average ethanol content (57.2%) compared to average ethanol content for the ABHRs without certification (48.8%). They also had a better percentage reduction score, averaging 89% against 78.9% for uncertified ABHRs ( Figure 9).

## Discussion

This study sought to assess and compare the quality of ABHRs in the Kenyan market before and during the pandemic period in order to build on knowledge and identify existing gaps in product quality status in the country. At the onset of the COVID-19 pandemic, the use of ABHRs gained popularity in Kenya and the world, leading to a ramp-up in production as several manufacturing companies repurposed their facilities for their production. The main publicized goal of ABHRs use was a quick (30 seconds) hand wash alternative to the traditional hand cleaning with water and soap, which has limited availability in low- and middle-income countries, which would ultimately improve pandemic containment strategies and overall infection prevention.

The number of available brands in the market during the pandemic progression period reduced (n = 55 to 35); this could be attributed to the increased surveillance by national regulatory bodies for compliant over-the-counter products leading to suspension and/or withdrawal of some brands in the Kenyan market. ^17^ The study saw a larger percentage of ABHRs with > log 2 efficacy performance. Despite these peri-pandemic products having NRA’s approval, 27.8% were found to have less than 90% bactericidal reduction activity as compared to 12.5% manufactured pre-pandemic. This could partially be due to reluctance by manufacturers in maintaining acceptable quality standards upon obtaining regulatory and standardization permits. Further to this, the study results found no significant relationship between total alcohol content and log reduction (r = -0.167, p = 0.480), studies have found that formulation of hand sanitizers play a major role in their effectiveness in antimicrobial action. ^18^ Some of the sanitizers tested in this study had acceptable levels of alcohol content but had lower log reduction factors, most likely due to wrong formulations which also affected the product pH. According to studies by Jing *et al*., 2020, ^19^ an antimicrobial efficacy performance of <1 log _10_ value is considered ineffective (no significant bactericidal activity), while undetectable level of bacterial growth is indicative of a higher antimicrobial efficacy than demonstrated. The findings of this study are consistent with findings from other studies that observed a lower logarithmic reduction factor of hand sanitizers during the pandemic period. A study in South Africa ^20^ observed that only 22% of the tested ABHRs were effective against the tested microorganisms.

The alcohols permitted for hand sanitizers production are ethanol, propanol, and isopropyl alcohol. The specification does not have an acceptance criterion for methanol. Methanol is controlled by the US FDA owing to its toxicity. Interim limits for methanol in ABHRs during the 2020 COVID-19 pandemic was 0.063% v/v up from the usual 0.02% v/v. ^21^^,^^22^ The US-FDA interim guidance has therefore been applied in the interpretation of the results for methanol content. Where substantial methanol concentration was recorded, it was interpreted as methanol substitution *i.e.*, methanol was used as the active ingredient. Methanol is toxic when absorbed in the body through the skin or ingested and can be life-threatening. Methanol substitution is a great public health concern due to the adverse health effects of methanol including metabolic acidosis, neurologic sequelae, and even death. Methanol substitution was found in 20% of the sanitizer brands with only 5% (n = 1) complying with FDA approval of <0.063% v/v methanol content. This is indicative of impure ethanol being used in the production process. The sum of the permitted alcohols was used for the decision statement on whether a product met the KEBS limit for alcohol content. Very few brands (25%) had over 60% alcohols content; however, none of the ABHRs met the 80% formulation guide set by the WHO. These findings are consistent with similar studies in Kenya ^23^ and South Africa ^24^ that reported presence of methanol impurities in 14.9% and 17% of the tested ABHRs respectively.

The shelf lives of the ABHRs in this study ranged from one to five years, which appeared to have been determined arbitrarily without any scientific basis on stability of the product. Some sanitizers did not declare the manufacture and/or expiration dates which poses a public health risk of use of expired and ineffective hand sanitizers. The pandemic saw a great improvement in the standard practice of appropriate labelling and listing ingredients and composition of products; however, four (4) brands had falsely listed the active ingredient. Results show that the product standardization mark had a slightly positive impact on the amount of ethanol used as well as improved log reduction scores, leading to better quality products which are capable of infection prevention and control. This proves that national regulatory bodies such as KEBS are efficient agents for enforcing industry standards. This study findings agree with findings by researchers from South Africa ^20^ who found that 41% of the ABHRs approved by South African Bureau of Standards (SABS), had less than 60% v/v alcohol content.

### Limitations of the study

a. Due to the high turnover rate of ABHRs brands in the market during the pandemic period, the study was unable to obtain the same brands for both pre- and peri-COVID-19 sanitizers
b. The number of sanitizers analyzed for alcohol content using gas chromatography wasn’t justifiably distributed. This is due to the cost implication relating to the test, which is outsourced, *versus* the limited budget.

## Ethical approval

This study was approved by the Scientific Ethical Review Unit (SERU) at KEMRI under protocol KEMRI/SERU/CBRD/226/4251.

## Author contributions

Conceptualization and project design, SO, JK (KEMRI) and FN (JKUAT); methodology, JK, SO and FN; formal analysis, JK, SO and FN; resources, SO and JK; data curation, SO.; writing—original draft preparation, JK, SO and FN; writing—review and editing, JK, SO and FN. All authors have read and agreed to the published version of the manuscript.

## Data Availability

Mendeley Data: ALCOHOL BASED HAND SANITIZERS IN KENYA- PRE AND PERI COVID-19, https://doi.org/10.17632/sj3dc9bw64.1 ^25^ This project contains the following underlying data:
- HAND SANITIZERS ANALYSIS RAW DATA- Blinded.xlsx (raw dataset) - WhatsApp Image 2021-03-04 at 15.28.50 (1).jpeg (Plate photo) - WhatsApp Image 2022-09-20 at 11.16.18.jpeg (Sample photo) Data are available under the terms of the Creative Commons Attribution 4.0 International license (CC-BY 4.0).
