# Supplementary material for: In vitro assessment and comparison of quality of alcohol-based hand rubs, pre- and peri-COVID-19 pandemic outbreak in Kenya
Source: F1000Res. 2024 May 9;12:1546. Originally published 2023 Dec 1. [Version 3] doi: 10.12688/f1000research.140226.3 (PMC11109557; doi:10.12688/f1000research.140226.3)
Supplement: Peer review history

# Reviewer report: Reviewer response for version 3

**DOI:** 10.5256/f1000research.165986.r276171

**Published:** 2024-06-27

**Authors:** Fred Tusabe

**Affiliations:** 1 Infectious diseases Institute, Kampala, Uganda

Before you narrow it to Kenya in the introduction, can you talk about various local ABHR production initiatives, especially in LMICs? Many studies have been published on this

1. Did you evaluate the stability of the components in the ABHR over time instead of relying solely on the branded information provided on the containers? For instance, if the ethanol concentration was 80% at the time of production, what was the concentration after one year, assuming that was the duration of the study? How much concentration was lost, etc.?
2. This statement isn't clear, ‘’ this could be attributed to the increased surveillance by national regulatory bodies for compliant over-the-counter products leading to suspension and/or withdrawal of some brands in the Kenyan market’’ did you intend to mean counterfeit products?

Is the work clearly and accurately presented and does it cite the current literature?

Partly

If applicable, is the statistical analysis and its interpretation appropriate?

Partly

Are all the source data underlying the results available to ensure full reproducibility?

Yes

Is the study design appropriate and is the work technically sound?

Partly

Are the conclusions drawn adequately supported by the results?

Partly

Are sufficient details of methods and analysis provided to allow replication by others?

Yes

Reviewer Expertise:

Infection prevention and control, WASH, AMR

I confirm that I have read this submission and believe that I have an appropriate level of expertise to confirm that it is of an acceptable scientific standard.

## References

[1] : Adequacy of alcohol-based handrub solution production practice in response to COVID-19 in public hospitals found in Addis Ababa, Ethiopia: a multicentered cross-sectional study. *J Pharm Policy Pract* .2021;14(1) : 10.1186/s40545-021-00321-y 39 10.1186/s40545-021-00321-y 33934722 PMC8088824

[2] : Comparison of Antibacterial Efficacy of Locally Produced Alcohol Based Hand Sanitizer and Commonly Available Commercial Hand Sanitizer Used in Healthcare Facilities in Uganda. *OALib* .2020;07(04) : 10.4236/oalib.1106221 1-13 10.4236/oalib.1106221

# Reviewer report: Reviewer response for version 3

**DOI:** 10.5256/f1000research.165986.r277250

**Published:** 2024-05-13

**Authors:** Kennedy Abuga

**Affiliations:** 1 Department of Pharmaceutical Chemistry, University of Nairobi, Nairobi, Kenya

Please view the review comments on the annotated PDF copy of the article found here.

Is the work clearly and accurately presented and does it cite the current literature?

Yes

If applicable, is the statistical analysis and its interpretation appropriate?

Yes

Are all the source data underlying the results available to ensure full reproducibility?

Yes

Is the study design appropriate and is the work technically sound?

Yes

Are the conclusions drawn adequately supported by the results?

Yes

Are sufficient details of methods and analysis provided to allow replication by others?

Yes

Reviewer Expertise:

NA

I confirm that I have read this submission and believe that I have an appropriate level of expertise to confirm that it is of an acceptable scientific standard.

# Reviewer report: Reviewer response for version 1

**DOI:** 10.5256/f1000research.153560.r255758

**Published:** 2024-04-22

**Authors:** Sandry Kesuma

**Affiliations:** 1 Pharmaceutical and food analysis, Health Polytechnic Ministry of Health Malang, Malang, East Java, Indonesia

**The whole article**

1. Some abbreviations suddenly appear in this article without the full term. Suggestion: Before using abbreviations throughout the article, please write the complete term in the first abbreviation, namely: Kenya Medical Research Institute (KEMRI), Kenya Bureau of Standards (KEBS), isopropyl alcohol (IPA), American Type Culture Collection (ATCC), Kenya Standard East Africa Standard (KS EAS), National Regulatory Authorities (NRAs)
2. Must be consistent in writing the terms pre-COVID-19 pandemic and peri-COVID-19 pandemic. Because in the Result section you only write pre-COVID-19, pre-pandemic, and peri-COVID-19 and peri-pandemic.

**p.1, Abstract- Results**

 The percentage of alcohol content you stated in the abstract is only for the 20 samples analyzed by GC-FID. If the analysis was performed on a total of 35 samples, the percentage results may differ. 

 Suggestion: state the total number of 20 samples analyzed for alcohol content in the abstract.

**p.4, Methods**- **Alcohol content assay and pH of alcohol-based hand sanitizers**

 Line 4,  The alcohol calibration standards were prepared by adding aliquots of pure alcohols - ethanol, IPA, methanol, n-propanol (n-PA) - to distilled water.

 The calibration standard states n-propanol (n-PA), but in p.4, Result- Declaration of active ingredients and Table 2. Analytical results for alcohol-based hand sanitizers, there is no information about n-PA and there is no column of n-PA analytical results in table 2.

 Suggestion: if you do not use n-PA in the calibration standard, then remove the n-propanol (n-PA) from the sentence.

**p.4, Methods**- **Alcohol-based hand sanitizers efficacy testing**

 Line 6, …200 mL of distilled as guided by WHO.

 Suggestion: ……200 mL of distilled water as guided by WHO

**p.6 Results- Alcohol content assay and pH of ABHRs**

 Only 20 samples were analyzed with GC-FID out of a total of 35 peri-COVID-19 pandemic samples.

 Suggestion: add an explanation of the reason "why only 20 selected samples were analyzed by GC-FID?"

**p.7, Result- Alcohol content assay and pH of ABHRs**

 The sample codes in the table (PBHR) are different from the sample codes discussed such as ABHR 09 and P-ABHR 36.

 Suggestion: match the use of example code

**p.7, Result- Alcohol content assay and pH of ABHRs**

 The pH value is a number that has no units. The pH scale is not an absolute scale. It is relative to a set of standard solutions whose pH is determined based on international agreement.

 Suggestion: it is not necessary to write "pH unit" after the pH value.[Refer Ref 1,2].

Is the work clearly and accurately presented and does it cite the current literature?

Partly

If applicable, is the statistical analysis and its interpretation appropriate?

Partly

Are all the source data underlying the results available to ensure full reproducibility?

Yes

Is the study design appropriate and is the work technically sound?

Partly

Are the conclusions drawn adequately supported by the results?

Partly

Are sufficient details of methods and analysis provided to allow replication by others?

Yes

Reviewer Expertise:

Pharmaceutical and food analysis, Pharmaceutical and food safety, chemical analysis

I confirm that I have read this submission and believe that I have an appropriate level of expertise to confirm that it is of an acceptable scientific standard, however I have significant reservations, as outlined above.

## References

[1] : Alcohol-Based Hand Sanitizer Combination of Eucalyptus Oil Extract against Aspergillus Niger Fungus. *Journal of Biomimetics, Biomaterials and Biomedical Engineering* .2023;63: 10.4028/p-8IgLe9 111-117 10.4028/p-8IgLe9

[2] : Hand sanitiser activity test of eucalyptus (Eucalyptus globulus) oil extract against Bacillus subtilis and Enterococcus faecalis bacteria. *Pharmacy Education* .2023;23(2) : 10.46542/pe.2023.232.179183 179-183 10.46542/pe.2023.232.179183

# Reviewer report: Reviewer response for version 1

**DOI:** 10.5256/f1000research.153560.r246475

**Published:** 2024-02-29

**Authors:** Fred Tusabe

**Affiliations:** 1 Infectious diseases Institute, Kampala, Uganda

In the introduction section, it is emphasized that hand washing with soap should always be accompanied by water.

 Throughout the paper, the term "alcohol-based handrubs (ABHRs)" has been interchangeably replaced with "alcohol-based sanitizer." Choose one for consistency.

 A citation is requested for the statement claiming that a 30-second application of alcohol-based hand rubs has superior disinfection efficacy compared to traditional soap and water approaches, resulting in more than a 3.5 log10 reduction in bacterial counts.

 The abbreviation ABHR is not an antimicrobial-based handrub.

 The statement regarding resistance to the use of alcohol-based hand rubs is noted, but without substantiation. While alcohol-based hand rubs are effective against various bacteria and viruses, they are primarily intended for hand hygiene rather than acting as general biocides. Therefore, citation 5, along with its corresponding statement, is considered misleading.

 Regarding the mode of action of alcohol-based hand rubs, it is acknowledged that there is no clear direct pathway for causing antimicrobial resistance. However, it is suggested that if poor-quality hand rubs become contaminated with resistant microorganisms during production or storage, repeated use of such products could contribute to the spread of resistant strains.

 Appreciation is expressed for providing literature on organisms that may be less susceptible to alcohol-based hand rubs. It is t it is not advisable to use alcohol-based hand rubs after visiting the latrine where *E. faecium* is likely to be acquired, or on visibly soiled hands where soap and water should be used.

 The importance of studies on emerging resistance due to poor-quality alcohol-based hand rubs is acknowledged. However, it is noted that in the current study, ATCC strains for *Escherichia coli *and *Staphylococcus aureus* were used as test organisms as the standards, but evidence of the efficacy of alcohol-based hand rubs on these strains in real-life scenarios is lacking.

 The title of the study needs to be rephrased since it was an in vitro study.

 In the methods section, clarification is needed on how the assessment for the declaration of active ingredients was conducted.

 The statement regarding the highly varied shelf lives allocated to the sanitizers requires elaboration or clarification.

 The first paragraph of the discussion section needs to be rephrased to clearly explain what the study did and its contribution.

 The discussion section should be rewritten to explicitly highlight how the findings of the study relate to or differ from others. Additionally, recommendations based on the findings should be provided.

Is the work clearly and accurately presented and does it cite the current literature?

Partly

If applicable, is the statistical analysis and its interpretation appropriate?

Partly

Are all the source data underlying the results available to ensure full reproducibility?

Yes

Is the study design appropriate and is the work technically sound?

Partly

Are the conclusions drawn adequately supported by the results?

Partly

Are sufficient details of methods and analysis provided to allow replication by others?

Yes

Reviewer Expertise:

Hand hygiene, Infection prevention and control, WASH

I confirm that I have read this submission and believe that I have an appropriate level of expertise to confirm that it is of an acceptable scientific standard, however I have significant reservations, as outlined above.

## References

[1] : Access to and Use of Hand Hygiene Resources during the COVID-19 Pandemic in Two Districts in Uganda, January-April 2021. *Am J Trop Med Hyg* .2023;109(4) : 10.4269/ajtmh.23-0031 881-889 10.4269/ajtmh.23-0031 37640292 PMC10551090

[2] : Comparison of Antibacterial Efficacy of Locally Produced Alcohol Based Hand Sanitizer and Commonly Available Commercial Hand Sanitizer Used in Healthcare Facilities in Uganda. *OALib* .2020;07(04) : 10.4236/oalib.1106221 1-13 10.4236/oalib.1106221

[3] : Establishment of district-led production of WHO-recommended Alcohol-Based Hand Rub (ABHR) during the COVID-19 pandemic: a model for improving access to ABHR during health emergencies. *Journal of Water, Sanitation and Hygiene for Development* .2023;13(10) : 10.2166/washdev.2023.143 847-856 10.2166/washdev.2023.143 PMC1089626238410156

[4] : Alcohol-Based Handrub Utilization Practice for COVID-19 Prevention Among Pharmacy Professionals in Ethiopian Public Hospitals: A Cross-Sectional Study. *Drug Healthc Patient Saf* .2021;13: 10.2147/DHPS.S295599 37-46 10.2147/DHPS.S295599 33623439 PMC7896735
